# Refractive-Index Profile Reconstruction in Graded-Index Polymer Optical Fibers Using Raman Spectroscopy

**DOI:** 10.3390/ma13102251

**Published:** 2020-05-14

**Authors:** Mikel Azkune, Angel Ortega-Gomez, Igor Ayesta, Joseba Zubia

**Affiliations:** 1 Department of Electronic Technology, Engineering School of Bilbao, University of the Basque Country (UPV/EHU), Plaza Ingeniero Torres Quevedo, 1, E-48013 Bilbao, Spain; 2 Department of Communications Engineering, Engineering School of Bilbao, University of the Basque Country (UPV/EHU), Plaza Ingeniero Torres Quevedo, 1, E-48013 Bilbao, Spain; angel.ortega@ehu.eus (A.O.-G.); joseba.zubia@ehu.eus (J.Z.); 3 Department of Applied Mathematics, Engineering School of Bilbao, University of the Basque Country (UPV/EHU), Plaza Ingeniero Torres Quevedo, 1, E-48013 Bilbao, Spain; igor.ayesta@ehu.eus

**Keywords:** polymer optical fibers (POF), Raman spectroscopy, graded-index fibers, refractive-index reconstruction

## Abstract

This work reports a novel method to create a 3D map of the refractive index of different graded-index polymer optical fibers (GI-POF), measuring the Raman spectra at different points of their transverse sections. Raman fingerprints provide accurate molecular information of the sample with high spatial resolution. The refractive index of GI-POFs is modified by adding a dopant in the preform; therefore, by recording the intensities of the Raman peaks related to the dopant material, a 3D map of the refractive index is rendered. In order to demonstrate the usefulness of the method, three different GI-POFs were characterized and the obtained results were compared with the information provided by the manufacturers. The results show accurate 3D maps of the refractive index taken in the actual GI-POF end faces, showing different imperfections that manufacturers do not take into account, such as the slight deviations of the azimuthal symmetry. The simplicity and the feasibility of the technique mean this method has high potential for fiber characterization purposes.

## 1. Introduction

In the last few decades, there have been continual advances in the performance of polymer optical fibers (POFs). As compared to glass fibers, POFs are easier and more economical to manufacture, safer to handle, and much more flexible [1,2,3,4]. There is a broad range of applications where POFs are excellent candidates, such as biosensing [5,6], structural health monitoring [7,8], optical amplification and lasing [9,10], and telemedicine and short-haul communications links [11]. Moreover, the high bandwidth of graded-index polymer optical fibers (GI-POFs) allows realization of high-speed information transmissions [4,12]. For all the aforementioned applications, and especially to define the bandwidth of GI-POFs, the refractive-index profile of these fibers must be controlled and measured [13].

There are two different principal measuring techniques in the literature that are used to characterize gradual refractive-index profiles: destructive and non-destructive techniques [14,15]. The disadvantages of destructive methods are obvious, while non-destructive methods (such as light focusing method, optical interference method, or methods based on the measurements of the bending of transverse rays) usually require the construction of highly accurate set-ups and the geometry of the samples must be perfectly circular or elliptical [16]. Moreover, all these techniques are mainly designed to characterize the refractive-index profile from the fiber preform and not from the fiber itself. The results obtained in this way may vary from the final distribution of the fiber, since the physical characteristics—even the index distribution—can be slightly different as a consequence of the stretching process in the manufacturing tower.

In this paper, we present a novel method to characterize the refractive-index distribution directly from GI-POFs based on Raman spectroscopy. Raman spectroscopy is a spectroscopic technique used to obtain information from molecular vibrations, in which the measured spectra provide an insight into both intramolecular information and intermolecular interactions, playing an important role in the elucidation of molecular structures [17,18]. In recent years, this spectroscopy technique has been widely used for different purposes [19,20,21], including for polymer characterization in POFs [22,23,24,25,26]. In our case, the Raman spectra are obtained at different points from the transverse section of GI-POFs, and we relate the proportion of some of the Raman peaks with the amount of dopants used to modify the refractive index. In this way, we can create 3D maps of the refractive-index distributions of the fibers. As far as we know, this is the first time that this kind of characterization has been carried out by using Raman spectroscopy. Moreover, the measurement system can be widely automated and the data analysis is straightforward, so the POF samples can be characterized in a very brief period of time, i.e., in a few minutes once the spectra are acquired.

The paper is organized as follows. First, the experimental set-up is described, together with the characteristics of the optical fibers employed. Then, the results for three different GI-POFs are shown. Finally, the main conclusions and potential applications are summarized.

## 2. Experimental Set-up

When a photon of wavelength $\lambda_{exc}$ strikes a molecule, both elastic and inelastic scattering occur. The elastically scattered photon has the same wavelength, while the inelastic photon has a different wavelength, which depends on the vibrational state of the molecules. As is well known, the Stokes Raman shift ∆ν is given by [27]:

(1) $$∆\nu \left(cm^{-1}\right)=\left[\frac{1}{\lambda_{exc}\left(nm\right)}-\frac{1}{\lambda_{m\to n}\left(nm\right)}\right]10^{7}$$

where $\lambda_{m\to n}$ is the wavelength of the Raman vibration band. The intensity of the scattered radiation $I_{m\to n}$ due to the vibrational transition m→n is:

(2) $$I_{m\to n}=N{\left(\nu_{exc}-\nu_{m\to n}\right)}^{4}\sum_{i,j}{\left|{\left(\alpha_{i}\right)}_{m\to n}E_{j}\right|}^{4}$$

where *N* is the number of molecules; $\nu_{exc}$ and $\nu_{m\to n}$ are the frequencies of the excitation laser and the Raman band, respectively; $\alpha_{i}$ is the polarizability of the molecule; and *Ej* is the electric field of the excitation.

Therefore, the Raman intensity is mainly affected by four factors: the light source irradiance through *Ej*, the excitation wavelength of the laser source $\nu_{exc}$, the scattering properties of the sample or scattering cross-section, and finally the concentration of the sample or the number of molecules in the measuring unit cell.

It is remarkable that the Raman intensity of a vibrational band depends on the number of active molecules *N*, as shown in Equation (2). If the vibration band corresponds to a certain doping molecule, the Raman intensity will be proportional to the number of this molecule. On the other hand, it is known that the refractive-index distribution is related to the concentration of doping molecules added during the polymerization process [28]. In the case of GI-POFs, the concentration is higher in the center of the fiber core and decreases gradually with radial distance. Taking into account these ideas, in this work an accurate and concise reconstruction of the refractive-index profile of GI-POFs has been carried out, with much higher accuracy than the mathematical modeling approaches. These mathematical models fail to quantify the deviation between the expected and actual refractive index value in the GI-POF, among other limitations. In order to overcome the drawbacks of the mathematical modeling and obtain a reliable refractive-index distribution in GI-POFs, we proposed a characterization technique based on Raman spectroscopy. This technique has the potential for non-destructive surface chemical analysis and allows us to obtain spatially well-defined fingerprints—or Raman peaks—with the chemical information of the sample.

### 2.1. Equipment

All the Raman measurements shown in this work were obtained using an InVia Raman confocal microscope from Renishaw (Gloucestershire, UK). In order to avoid any fluorescence, the primary excitation source used was a 785 nm laser. The optical power on the sample was 7.64 mW. This power is low enough to avoid any thermal effect or material degradation. Besides, we used a pinhole in the launching path to reduce the spot dimension, therefore increasing the lateral resolution. Depending on the GI-POF, the magnification of the microscope objective was 50× (spot size = 2 mm) or 20× (spot size = 7 mm). Additionally, all measurements were done in high confocality mode, so we were able to collect the chemical information of the end face of the fibers. The grating was at a rate of 1200 lines/mm and the acquisition time for each spectrum was ten seconds. For this grating, according to the manufacturer the spectral resolution is 0.5 cm^−1^.

### 2.2. Sample Preparation

**Figure 1:**
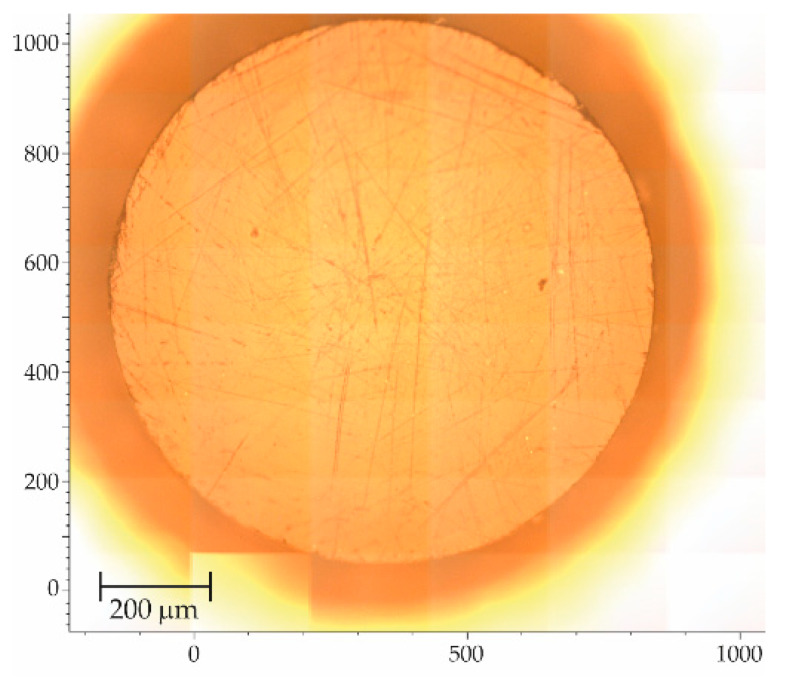
Microscope image composition of a polymer optical fiber (POF) sample obtained from the Renishaw InVia Raman microscope using a 20× objective.

All used GI-POF samples were 10 cm in length, although the fiber length does not influence the measurement because the active Raman depth (a few microns) is negligibly small compared to the fiber length. First, the end faces of the GI-POF samples were properly polished using different grain sandpapers and by rinsing them in isorpopilyc alcohol, in order to get repetitive measurements and remove all possible impurities that could mask or perturb the spectra. Then, the samples were placed on a motorized stage with a step resolution of 1 μm. Afterwards, we took a wide image of the end face in the Raman microscope in order to launch the mapping experiment, as can be seen in Figure 1.

### 2.3. Measurements Set-Up

**Figure 2:**
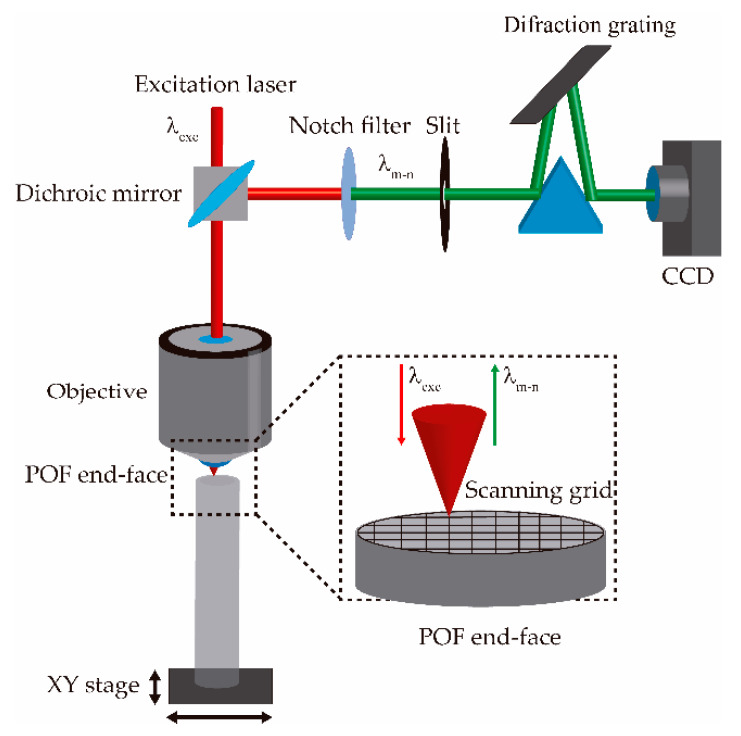
Set-up used in all of the experiments, based on a typical Raman set-up, where the scanning grid is performed on the graded-index polymer optical fibers (GI-POF) end face.

Subsequently, we divided the GI-POF end face into a grid of hundreds of points where the Raman spectra would be recorded (Figure 2). At each point, we recorded the average of three consecutive spectra acquisitions. Once the Raman spectra measurements were done, we removed the baseline using a 4S FillPeaks algorithm [29] for all mapped spectra. Afterwards, we smoothed them using a Savitzky–Golay second-order filter with 21 points, using an ad hoc script in R.

### 2.4. Data Procesing and 3D Map Reconstruction

In order to process the recorded Raman spectra during the mapping, first we defined the Raman band, whose intensity increases as the doping concentration increases, i.e., as a function of the radial distance to the fiber axis (*Dopant_Peak_*). The choice of the active Raman band was conducted by comparing the Raman spectra at the core axis and at the core extreme limit. Furthermore, in order to set an internal standard peak, we selected the reference intensity from the well-known Raman band assigned to the substrate material, which does not correspond to the doping material, meaning it does not change through the core area (*Substrate_Peak_*). We defined the Raman intensity ratio as follows:

(3) $$R=\frac{I\left(Dopant_{Peak}\right)}{I\left(Subsrate_{Peak}\right)}$$

Once the ratio was calculated, the next step was to calculate the predicted value of the refractive index ($n_{calculated})$, following the next expression:

(4) $$n_{calculated}=R*\frac{\Delta \left(n_{GI-POF}\right)}{\max\left(R\right)}+min\left(n_{GI-POF}\right)$$

where max(R) is the maximum ratio value of the whole map; $min\left(n_{GI-POF}\right)$ is the minimum refractive-index value in the GI-POF, also called *n*_2_; and $\Delta \left(n_{GI-POF}\right)$ is the difference between the maximum and minimum of the refractive indexes in the GI-POF. Plotting the value of the $n_{calculated}$ in each of the measured points, we rendered the refractive-index reconstruction and built a 3D map.

On the other hand, the current GI-POF’s refractive-index profile is usually expressed with the mathematical expressions described in Equations (5) and (6) [30]:

(5) $$n\left(r\right)=n_{1}{\left[1-2∆{\left(\frac{r}{a}\right)}^{g}\right]}^{\frac{1}{2}},\;0\leq r\leq a$$

(6) $$n\left(r\right)=n_{2},\;r>a$$

where *n*_1_ and *n*_2_ are the refractive indexes of the center axis and the cladding, respectively; *a* is the core radius; *g* is the index exponent that is the parameter of the index profile; and ∆ is the relative index difference, defined as follows:

(7) $$∆=\frac{n_{1}^{2}-n_{2}^{2}}{2n_{1}^{2}}$$

These expressions were used in this work in order to model the GI-POF refractive index distributions. We compared them with the results obtained from Equation (4).

### 2.5. Studied GI-POFs

In order to demonstrate the usefulness of this method, it was employed with three different GI-POF manufacturers. The main features of the employed fiber samples are summarized in Table 1. The reason for measuring those fibers was to prove the versatility of the presented method in commercial and non-commercial GI-POFs with different characteristics, such as fiber diameter or dopant material.

As can be observed in Table 1, we analyzed two PMMA-based GI-POFs, one of which was made from Cytop^TM^. The first one showed a 1-mm wide diameter and was purchased from FiberFinn. This GI-POF was fabricated for short-range communication purposes and the dopant used for refractive index modification was benzyl benzoate [31]. The second fiber was a Cytop^TM^-based GI-POF called Lucina^TM^. Cytop^TM^ is the registered trademark of a fluoropolymer from Asahi Glass. It has a low refractive index and high optical transparency because it is C-H-bond-free and the C-F bond is transparent in the range of 650–1300 nm. It was conceived for high-capacity communications, as it shows an attenuation below 60 dB/Km at 1100 nm, outstripping the first option.

The third GI-POF we studied was the experimental fluorescence GI-POF (EFGI-POF) fiber, which was also based on PMMA and used the same dopant to control the refractive index. This fiber was fabricated for research purposes, which was the main reason why Rhodamine 6G molecules were also incorporated [9]. As already pointed out, the measurements were performed using a 785 nm laser in order to avoid any absorption or emission effects from the Rhodamine 6G.

## 3. Results and Discussion

### 3.1. FiberFinn GI-POF

**Figure 3:**
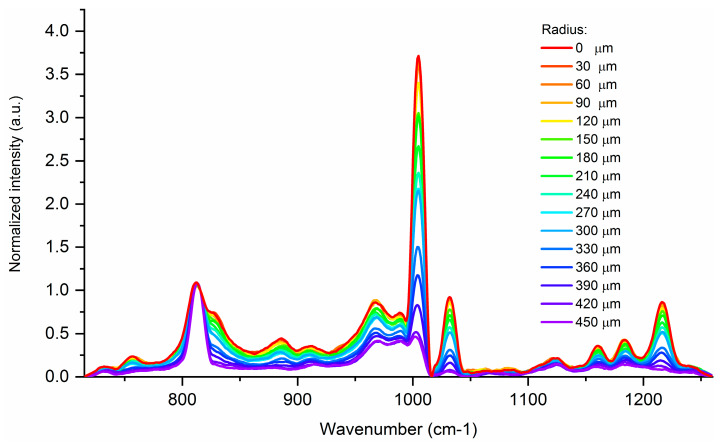
Normalized Raman spectra in a radial direction of the FiberFinn GI-POF.

The first analyzed GI-POF was manufactured by FiberFinn. This 1-mm diameter fiber is made from PMMA and the refractive-index profile is obtained by adding a fluorinate dopant. Prior to measuring all the spectra of the end face, we recorded different Raman spectra in a defined radial direction in order to determine the Raman peaks corresponding to the PMMA and to the dopant. The obtained results, which were normalized to a well-known PMMA peak placed at 815 cm^−1^, are shown in Figure 3 [26].

**Figure 4:**
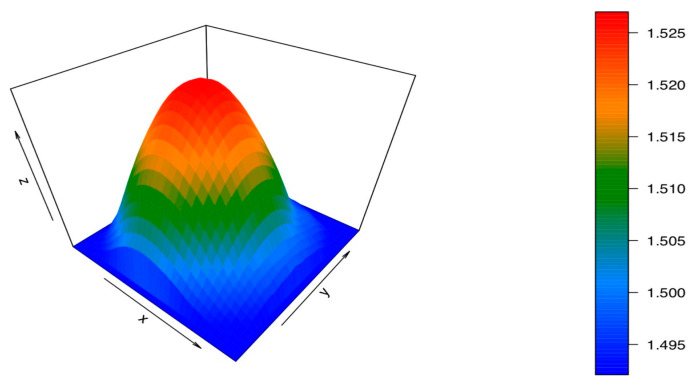
The 3D refractive-index reconstruction of the FiberFinn GI-POF.

From the obtained results, it can be seen that the peak placed at 1006 cm^−1^ expresses the highest variation in the peak intensity with the radial distance. This peak is the main Raman peak of benzyl benzoate, the dopant used for the refractive-index modification [31]. Therefore, this peak was set as the dopant peak in Equation (3), and the peak placed at 815 cm^−1^ was set as the substrate peak. Then, we performed the end face mapping. In order to render a 3D refractive-index map, we followed the steps described in previous sections, using a 30 μm step resolution and a 20× objective. The reconstruction is shown in Figure 4.

**Figure 5:**
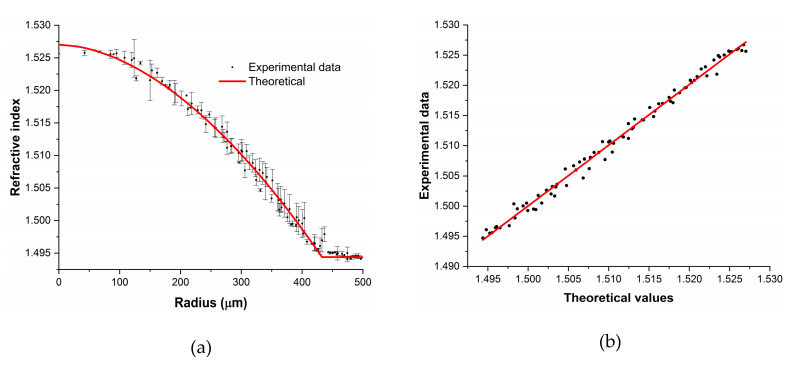
(**a**) Calculated mean refractive-index values (black dots with error bars) of the FiberFinn GI-POF and the refractive-index distribution provided by the manufacturer (red solid line). (**b**) Regression of both distributions.

From the reconstruction, we could state that the GI-POF profile of this fiber follows the expected shape with negligible imperfections. In order to visualize slight imperfections of the refractive index in the GI-POF, we display the mean *n_calculated_* and the standard deviation for each radial distance, as well as the refractive-index value provided by the manufacturer (see Figure 5). Additionally, the regression of the measured refractive-index distribution is also plotted, along with the one provided by the manufacturer.

Figure 5a shows the refractive-index profile obtained averaging all the spectra corresponding to different azimuthal angles for each radial distance, as well as the refractive index profile provided by the manufacturer. Hence, the standard deviation for each radius represents the azimuthal asymmetry of the fiber, which was 0.2% in the worst case. As can be seen, these standard deviation values mean that for the same radius the actual refractive index can be different, something that is not taken into account in traditional refractive-index measurements and that dictates the quality of the GI-POF. Additionally, Figure 5b shows that the overall shape of the calculated refractive index follows the distribution provided by the manufacturer. According to the regression plot, the relation between the measured and manufacturer´s reported values is linear, with a Pearson’s r^2^ value of 0.985, concluding that the method is valid for these types of measurements.

### 3.2. Lucina^TM^

**Figure 6:**
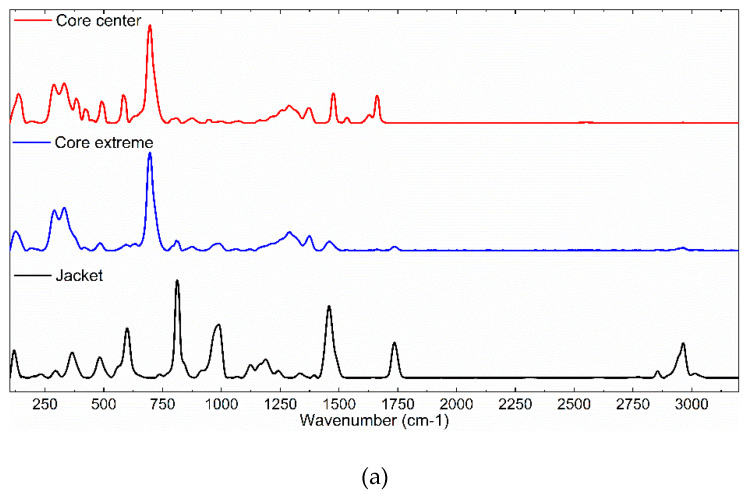

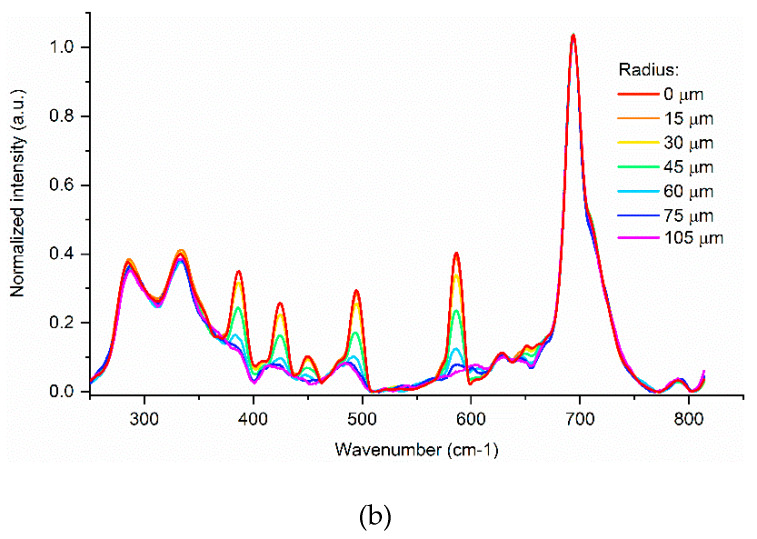
(**a**) Raman spectra of Lucina^TM^ GI-POFs obtained from different sections (core center, core extreme, and jacket). (**b**) Normalized Raman spectra in radial direction.

The second analyzed GI-POF was the Lucina^TM^ fiber. Both substrate and dopant materials differed from those of the previous fiber. The substrate material in this case is Cytop^TM^, while the manufacturer does not provide information about the dopant employed to modify the refractive index. Therefore, we first recorded Raman spectra from different fiber locations in order to choose the most interesting peaks to record. The obtained spectra are shown in Figure 6.

**Figure 7:**
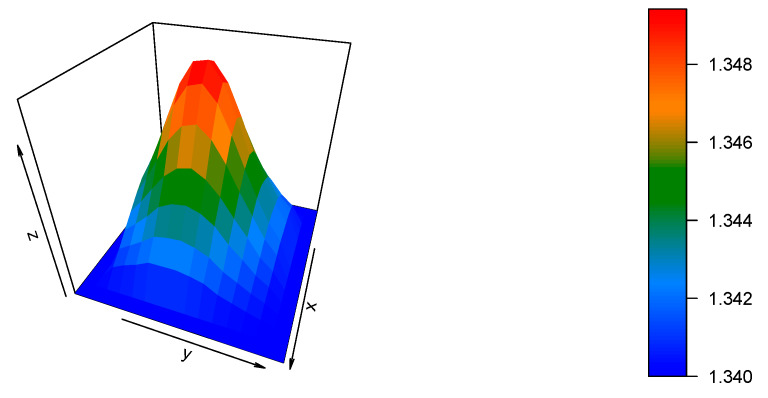
The 3D refractive-index reconstruction of the Lucina^TM^ GI-POF.

As can be seen in Figure 6, the dopant shows many Raman bands. Additionally, from the spectra recorded from the jacket, we can conclude that the manufacturer made this section with PMMA, with the possible aim of providing mechanical protection. The wavelength shifts for the dopant and the reference were set at 590 and 695 cm^−1^, respectively. For this GI-POF, we set the 50× objective, as the fiber diameter was significantly smaller (i.e., 120 μm). The spectra were recorded with steps of 15 μm. The rendered 3D map is shown in Figure 7.

**Figure 8:**
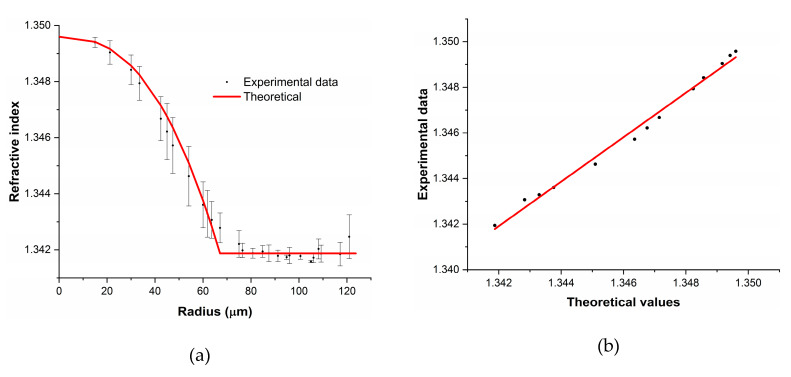
(**a**) Calculated mean refractive-index values (black dots with error bars) of the Lucina^TM^ GI-POF and the refractive-index distribution provided by the manufacturer (red solid line). (**b**) Regression of both distributions.

The refractive-index reconstruction shown in Figure 7 is noticeably different in comparison to the previous case—the overall shape is sharper. Additionally, the refractive-index values are rather small, which was an expected change, as both the substrate and dopant materials are CYTOP-based materials. Following the analysis of the previous case, the calculated refractive-index profile, the refractive index provided by the manufacturer, and the regression are displayed in Figure 8.

For this case, it can be observed that the azimuthal asymmetry of the refractive index is smaller than in the FiberFinn case, being 0.08% in the worst case. However, the refractive index of the modeling fits better for this fiber, as the mean values of the calculated refractive indexes are almost placed on the modeled points. Regarding the regression, we conclude that this calculation method is also validated for this fiber, as the regression is linear for refractive-index values of the core (i.e., refractive-index values higher than 1.3242), with a Pearson’s r^2^ value of 0.986.

### 3.3. EFGI-POF

**Figure 9:**
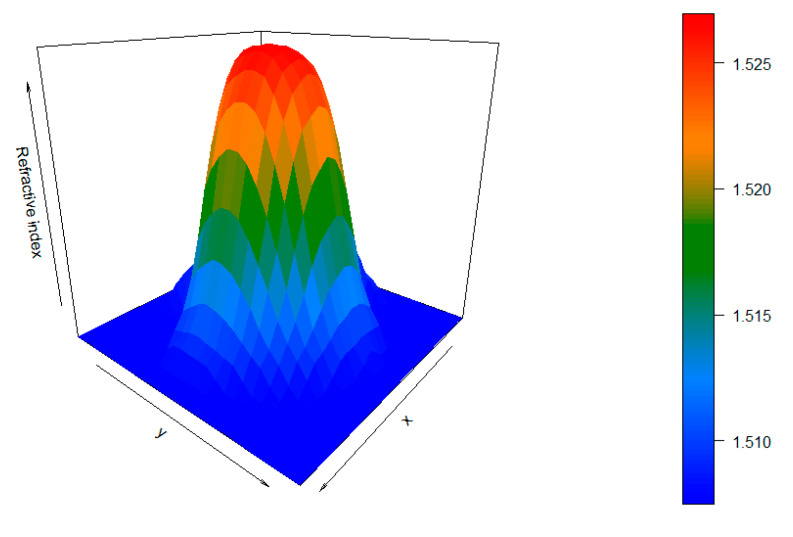
The 3D refractive-index reconstruction of the experimental fluorescence GI-POF (EFGI-POF).

The last fiber we analyzed had similar characteristics to the one manufactured by FiberFinn. For instance, the substrate and dopant materials, as well as the fiber diameter, were the same as the first case. However, this fiber had the peculiarity of the organic dye Rhodamine 6G being added during the fabrication process. This dye is well characterized within the literature and its Raman peaks do not interfere with either the dopant or the substrate material peaks. However, the fluorescence effect of the dye could hide the Raman peaks if an incorrect laser source was used, such as the typical 532 nm light source. Therefore, the mapping was recorded using the same parameters as in the first case. The 3D plot is shown in Figure 9.

**Figure 10:**
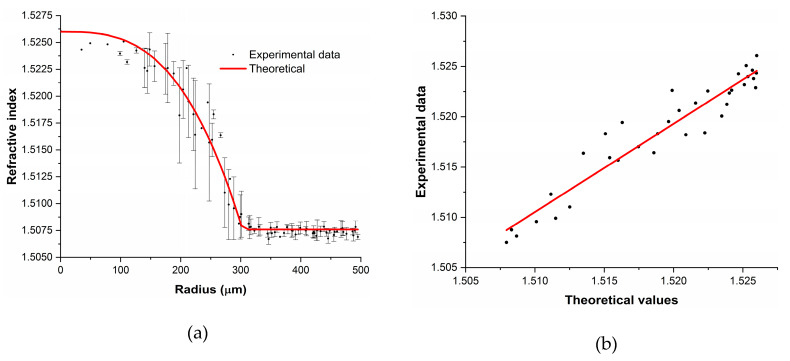
(**a**) Calculated mean refractive-index values (black dots with error bars) of the EFGI-POF and the refractive-index distribution obtained from the modeling (red solid line). (**b**) Regression of both distributions.

The area with the graded-index distribution is not as wide as the core diameter, with the radius of the graded area being 300 μm. In the same way as for the previous cases, in Figure 10 we compare the values of the calculated refractive indexes with the ones obtained from the modeling, as well as the regression between them.

From Figure 10a we can observe that the refractive index varies at different points of the fiber with the same radius, with the azimuthal asymmetry being 0.36% in the worst case. This shows that the quality of the fiber is not as high as in the two previous samples. Figure 10b shows a regression with a higher dispersion than the previous cases, linked with the poorer quality of the refractive-index profile of this GI-POF (r^2^ = 0.972). It is also remarkable that the fluorescent dye added to the fiber does not interfere with the measurements, which was expected due to the high molecular selectivity of the Raman spectroscopy.

Considering the wide range of the presented GI-POF cases, the versatility of the technique is proven, as it is capable of reconstructing 3D maps of the refractive index of each sample. Additionally, other metrics are also calculated, such as the azimuthal asymmetry or the difference between the calculated refractive index and the value provided by the manufacturers, showing all of the imperfections of the refractive index. The accuracy of these metrics could determine the quality and feasibility of the GI-POFs for the desired application.

## 4. Conclusions

A novel method for reconstructing the refractive-index profile in GI-POFs is presented. This method is based on Raman spectroscopy mapping of the surface of the GI-POFs and has been validated with three different GI-POFs—two of the GI-POFs are based on PMMA and the other is based on Cytop^TM^. Moreover, these fibers had different dopants in order to obtain the desired graded-index profile, and one of them had a fluorescent organic dye added into the fiber core. For each of the three GI-POFs, a 3D map of the refractive-index was reconstructed and compared to the mathematical modeling provided by manufacturers. The variation of the refractive index per radius and the regression between the calculated and the model were also analyzed, showing that actual GI-POFs have azimuthal asymmetries, which were quantified. Thus, the presented method shows good potential in terms of GI-POF characterization, which is a critical parameters for high data capacity communications.

## Figures and Tables

**Table 1:** Main features of the analyzed GI-POFs

Name	Company	Core Diameter	Attenuation (dB/km)	Polymer
OMG-Giga-SE100	FiberFinn	1 mm	<200 @ 650 nm	Poly(methyl methacrylate) (PMMA)
Lucina^TM^	Asahi Glass	125 m	<30 @ 1200 nm	CYTOP^TM^
Experimental Fluorescence GI-POF		1 mm	<200 @ 650 nm	PMMA + 12 ppm Rhodamine 6G
